# Vitamin D as an intervention for improving quadriceps muscle strength in patients after anterior cruciate ligament reconstruction: study protocol for a randomized double-blinded, placebo-controlled clinical trial

**DOI:** 10.1186/s13063-024-08094-w

**Published:** 2024-04-11

**Authors:** Michael Tim-yun Ong, Xiaomin Lu, Ben Chi-yin Choi, Siu-Wai Wan, Qianwen Wang, Gene Chi-wai Man, Pauline Po-yee Lui, Daniel Tik-Pui Fong, Daniel Kam-wah Mok, Patrick Shu-hang Yung

**Affiliations:** 1 Department of Orthopaedics and Traumatology, Faculty of Medicine, The Chinese University of Hong Kong, Room 74029, 5/F, Lui Che Woo Clinical Science Building, Prince of Wales Hospital, Shatin, Hong Kong SAR, China; 2 Department of Food Science and Nutrition, The Hong Kong Polytechnic University, TU314, Block U, Hung Hom, Hong Kong SAR, China

**Keywords:** Vitamin D, Quadriceps strength, Anterior cruciate ligament (ACL), Anterior cruciate ligament reconstructions (ACLR)

## Abstract

**Background:**

The goal of anterior cruciate ligament reconstruction (ACLR) is to restore the preinjury level of knee function to return to play (RTP). However, even after completing the rehabilitation programme, some patients may have persistent quadriceps muscle weakness affecting knee function which ultimately leads to a failure in returning to play. Vitamin D has been long recognized for its musculoskeletal effects. Vitamin D deficiency may impair muscle strength recovery after ACLR. Correcting vitamin D levels may improve muscle strength.

**Methods:**

This is a double-blinded, randomized controlled trial to investigate the effects of vitamin D supplementation during the post-operative period on quadriceps muscle strength in anterior cruciate ligament (ACL)-injured patients. Patients aged 18–50 with serum vitamin D < 20 ng/ml, unilateral ACL injury, > 90% deficit in total quadriceps muscle volume on the involved leg compared with uninvolved leg, Tegner score 7 + , and no previous knee injury/surgery will be recruited. To assess patient improvement, we will perform isokinetic and isometric muscle assessments, ultrasound imaging for quadriceps thickness, self-reported outcomes, KT-1000 for knee laxity, biomechanical analysis, and Xtreme CT for bone mineral density. To investigate the effect of vitamin D status on quadriceps strength, blood serum samples will be taken before and after intervention.

**Discussion:**

Patients with low vitamin D levels had greater quadriceps fibre cross-sectional area loss and impaired muscle strength recovery after ACL. The proposed study will provide scientific support for using vitamin D supplementation to improve quadriceps strength recovery after ACLR.

**Trial registration:**

ClinicalTrials.gov NCT05174611. Registered on 28 November 2021.

**Supplementary Information:**

The online version contains supplementary material available at 10.1186/s13063-024-08094-w.

## Administrative information

Note: The numbers in curly brackets in this protocol refer to the SPIRIT checklist item numbers. The order of the items has been modified to group similar items (see http://www.equator-network.org/reporting-guidelines/spirit-2013-statement-defning-standard-protocol-items-for-clinical-trials/).

**Table:** 

Title {1}	Vitamin D as an intervention for improving quadriceps muscle strength in patients after anterior cruciate ligament reconstruction: study protocol for a randomized double-blinded, placebo-controlled clinical trial
Trial registration {2a and 2b}	Clinical.Trials.gov identifier: NCT05174611. 28 November 2021
Protocol version {3}	Original version dated: 8 December 2022 First revision date: 7 March 2023 • Primary reason for revision: Amendment suggested by the ethics committee for the further justification of the required sample size
Funding {4}	Funded by Direct Grant, The Chinese University of Hong Kong (Ref: 2021.037) The funder has no role in the design of the study; collection, analysis, and interpretation of the data; and writing of the manuscript
Author details {5a}	Michael Tim-yun Ong^1*^, Xiaomin Lu^1*^, Ben Chi-yin Choi^1^, Qianwen Wang^1^, Daniel Kam-wah Mok^2^, Gene Chi-wai Man^1^, Pauline Po-yee Lui^1^, Daniel Tik-Pui Fong^1^, Patrick Shu-hang Yung^1^ ^1^Department of Orthopaedics and Traumatology, Faculty of Medicine, The Chinese University of Hong Kong, Hong Kong SAR, China ^2^Department of Food Science and Nutrition, The Hong Kong Polytechnic University, Hong Kong SAR, China
Name and contact information for the trial sponsor {5b}	The Chinese University of Hong Kong Department of Orthopaedics and Traumatology Dr. Michael Tim Yun Ong Rm 74,034, 5/F, Lui Che Woo Clinical Science Building, Prince of Wales Hospital, Shatin, Hong Kong SAR, China michael.ong@cuhk.edu.hk
Role of sponsor {5c}	Role of study sponsor: role and ultimate authority in study design; collection, management, analysis, and interpretation of the data; writing of the report; and the decision to submit the report for publication Role of study funder: no roles in the collection, management, analysis, and interpretation of the data; writing of the report; or the decision to submit the report for publication

## Background and rationale {6a}

In Hong Kong, more than 3000 anterior cruciate ligament reconstructions (ACLR) are carried out annually to restore knee function after an ACL injury. The primary aim of ACLR, particularly for athletes, is to return to sports and recondition the athlete to their pre-injury level of sport. Despite successful surgery and a rigorous rehabilitation process, some athletes still fail to meet the return-to-play (RTP) criteria. Additionally, 23% of those who returned to high-risk sports suffered a second ACL injury [21].

After ACLR, the patient would undergo post-operative rehabilitation to strengthen the knee muscles. Most of the patients would be expected to RTP by 12 months. Despite a comprehensive rehabilitation programme, a systematic review showed that up to 35% of patients would fail to return to their preinjury level of sport [1].

Patients can experience substantial quadriceps muscle weakness after ACLR which can persist for over 1 year after surgery. Quadriceps muscle weakness after ACLR is a major limiting factor for functional recovery. It is contributed by arthrogenic muscle inhibition and muscle atrophy, which result from muscle disuse, joint swelling, pain, inflammation, and damage of neuroreceptors in the joint after the surgery. Muscle size is a determinant of muscle strength. Post-operative quadriceps muscle atrophy is inevitable, but muscle strength should be regained through rehabilitation. As the patient continues to exercise during rehabilitation, the atrophic response subsides and the strengthening training stimulates significant hypertrophy in the quadriceps muscle, thus increasing the quadriceps strength [6]. However, in some patients, this hypertrophic response is insufficient and can lead to persistent quadriceps muscle atrophy after ACLR.

In recent years, there has been a surge of interest in the research field investigating vitamin D status in athletes and examining its musculoskeletal effects. Vitamin D can be obtained from UV-B rays in sunlight or diet. There are 2 major compounds in vitamin D, which are vitamin D2 and vitamin D3. 25-Hydroxyvitamin D3, the active form of vitamin D3, can increase protein synthesis and the number of type II muscle cells by binding to Vitamin D receptors [12], which in turn leads to improved muscle strength and contraction velocity. Vitamin D deficiency can cause a reduction in type II muscle fibres, negatively affecting muscle function and leading to proximal muscle weakness.

In 2011, a study was conducted to examine the relationship between serum vitamin D levels and the isometric knee extension test in patients with ACL injury [3]. The study observed patients at the pre-op stage and 3 months after ACLR. The results showed that patients with sufficient levels of vitamin D had significant improvement in the injured side at the post-op stage as compared to their pre-op stage. However, no significant improvement was found in patients with vitamin D insufficiency. Additionally, patients with vitamin D sufficiency had a significantly greater percentage of changes in isometric peak torque as compared to those with insufficiency. This study suggests that patients with vitamin D insufficiency may have impaired muscle strength recovery following their ACLR.

This study would benefit patients who suffered from persistent quadriceps weakness and atrophy after ACLR, thus improving the post-operative outcome. This study aims to conduct a double-blinded, randomized controlled trial to examine the therapeutic effects of vitamin D supplements on improving quadriceps muscle strength in patients with quadriceps muscle weakness after ACL reconstruction. The investigators hypothesize that vitamin D supplements will improve the quadriceps muscle weakness and size of patients with vitamin D deficiency after ACLR.

## Objectives {7}

This study aims to conduct a double-blinded, randomized controlled trial to examine the therapeutic effects of vitamin D supplements on improving quadriceps muscle strength in patients with quadriceps muscle weakness after ACLR.

## Trial design {8}

This study is a randomized, double-blinded, placebo-controlled clinical trial to investigate the effects of vitamin D supplements for patients with quadriceps muscle weakness after ACLR. The intervention group receives 2000 IU vitamin D_3_ per day for 16 weeks whereas the control group receives a placebo.

Data is assessed at the four measurement time points from the participants:

1. Baseline/inclusion
2. First follow-up: 8 weeks after intervention commencement
3. Second follow-up: 16 weeks after intervention commencement
4. Third follow-up: post-intervention at 2 months

## Methods: participants, interventions, and outcomes

### Study setting {9}

Patients are recruited from a local hospital in Hong Kong. These patients will be followed up at the Orthopaedics Outpatient Clinic at the Prince of Wales Hospital for clinical examination and questionnaire filling. While for the muscle assessments, it will be conducted at the Sports Medicine and Rehabilitation Centre at the Chinese University of Hong Kong Medical Centre.

### Eligibility criteria {10}

The inclusion criteria are as follows:

1. Aged 18–50 with unilateral ACL injury
2. Sporting injury with a Tegner score of 7 + 
3. Serum D level remained < 20 ng/ml
4. Limb symmetry index of isokinetic quadriceps strength < 90% in the injured leg of the contralateral leg
5. Both knees without a history of injury/prior surgery

The exclusion criteria are as follows:

1. Concomitant bone fracture, major meniscus injury, or full-thickness chondral injuries requiring altered rehabilitation programme post-operatively
2. Pre-operative radiographic signs of arthritis
3. Metal implants that would cause interference on MRI
4. Non-HS graft for ACLR
5. Patient non-compliant with the rehabilitation programme
6. Regular sunbed users

### Who will take informed consent? {26a}

Trained research assistants will obtain written informed consent from all participants prior to their participation in this study. Our research assistants will first explain to eligible participants our programme in detail. The study will be carried out in compliance with the Declaration of Helsinki and the ICH-GCP. Ethical approval will be obtained from the local IRB before the study starts. Informed consent must be obtained from all patients in order to participate in the study.

### Additional consent provisions for collection and use of participant data and biological specimens {26b}

Consent of participant data and biological specimens are also included in the informed consent. Blood specimens in this study will be disposed of after testing and will not be used for genetic analysis or used in other studies. Every participant will be represented by an ID number, and personal information such as name, address, and telephone number will be kept confidential before, during, and after the study.

### Interventions

#### Explanation for the choice of comparators {6b}

Patients randomized to the control arm will receive a placebo with the same appearance as vitamin D_3_. As a placebo will look and taste like vitamin D_3_, this can ensure the participants are blinded to the treatment. The utilization of a placebo that closely mimics the appearance and flavour of vitamin D3 will effectively blind the participants to their treatment.

#### Intervention description {11a}

For the proposed study, we will use 2000 IU/day as advised by the Endocrine Society [10]. A previous study has shown that 2000 IU/day for 4 months showed significant improvement in muscle strength [22]. Therefore, for the proposed study, we will use 2000 IU/day for a duration of 16 weeks. Subjects will be randomized into two study groups: (1) placebo group—patients receive placebo; (2) intervention group—patients receive a daily dose of 2000 IU of vitamin D3 supplements. Supplements will be dispensed to participants in baseline and 1st follow-up to increase the subject compliance.

All study tablets including the supplement and placebo will be manufactured according to the Good Manufacturing Practice (GMP) guidelines for quality assurance. They will be taken with water at breakfast, one tablet at a time and once daily. The treatment will last for 16 weeks after which the supplementation will be stopped. Subjects will continue with their usual lifestyles in diet and physical activity without receiving any other treatment for muscle health.

#### Criteria for discontinuing or modifying allocated interventions {11b}

Vitamin D has been considered safe for users when taken in appropriate doses [13]. Participants will be advised that in the event of nausea, vomiting, muscle weakness, or confusion, they should discontinue taking supplements and seek medical attention. The medical staff will perform the evaluation determining if immediate removal from the study is necessary for their best interest to safeguard their health.

#### Strategies to improve adherence to interventions {11c}

Patients will be contacted weekly for their intake of the intervention and 1 week before the assessment to enhance the attendance rate. Special assessment session on the weekend or in the evening will be arranged under special circumstances to enhance subject compliance. Patients who default a scheduled appointment will be contacted by the investigators to re-arrange another appointment within 1 week. In the case of patient non-compliance, study personnel would remove the patient from the study only when multiple failed attempts to contact and convince the patient occur. If the patient chooses to withdraw from the study before the end of the study period, the reason and termination date will be recorded. As far as possible, we will invite patients who would like to withdraw to attend the final assessment.

#### Relevant concomitant care permitted or prohibited during the trial {11d}

Participants remain on their standard treatment and medication procedures throughout the study period, and clinicians are advised to manage participants in the usual manner subject to the caveats outlined above.

#### Provisions for post‑trial care {30}

Not applicable, since ancillary and post-trial care is provided within the standard care.

### Outcomes

The primary objectives of this study are to track the changes in peak torque and fatigue index (FI) of isokinetic muscle strength over a period of 6 months. The peak torque, measured in Newton metre (Nm), will be the highest recorded value among the 30 repetitions during the isokinetic muscle strength test, and the FI will be utilized to determine the per cent decrease for each variable that correlates with muscle endurance. Secondary outcome measures include (1) assessment of isometric muscle strength through quadriceps rate of torque development (RTD) and central activation ratio (CAR), (2) quadriceps muscle volume and muscle thickness, (3) evaluation of serum 25(OH)D using LC-Qtrap/MS, (4) measurement of passive knee laxity using KT-1000 knee ligament arthrometer (MEDmetric Corp., San Diego, CA, USA), (5) analysis on the reaming size of the bone using XtremeCt II, (6) the evaluation of ground reaction force will be carried out using a synchronized force plate located at the centre of the capture volume at 1000 Hz, (7) assessment of knee joint moments will be assessed by the skin marker-based motion analysis system, (8) distance by single-leg hop test, and (9) self-reported outcome assessing pain, disability, and activity level on knee function will be evaluated during the 6 months of follow-up.

### Participant timeline {13}

**Fig. 1:**
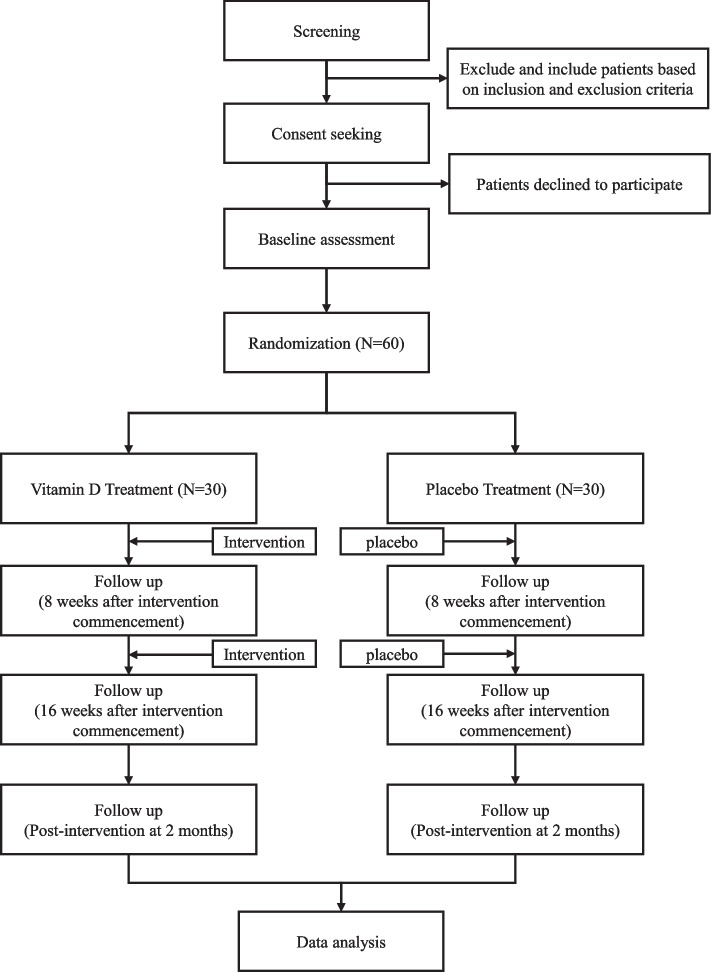
Study flowchart

The SPIRIT reporting guidelines were used to ensure the completion of the study protocol (Additional file 1) [4]. The study flowchart is illustrated in Fig. 1.

### Sample size {14}

Quadriceps muscle strength will be employed as the primary outcome for sample size estimation. As reported [22], the difference in quadriceps between the two groups is 0.88. It is estimated that a sample size of 28 in each group will have 90% power to detect a significant difference using a two-sided independent *t* test with a 0.05 significance level (G*Power 3.1.9.4). Taking account of the 20% dropout rate, we further increase the sample size to *n* = 30 for each arm (total *n* = 60).

### Recruitment {15}

Patients who have persisting quadriceps muscle weakness after ACLR will be recruited consecutively.

The centre staff will assist in identifying eligible patients from the Department of Orthopaedics and Traumatology at Prince of Wales Hospital, Hong Kong, based on the inclusion and exclusion criteria and send them to us for screening. These patients will be screened at the Sports Medicine and Rehabilitation Centre at the Chinese University of Hong Kong Medical Centre. The patients will be explained the study procedures by the principal investigator. Patients who consent to participate in the study will attend a scheduled visit for baseline examination. The completion of the trial is expected to take 36 months.

### Assignment of interventions: allocation

#### Sequence generation {16a}

##### Randomization and blinding

A total of 60 patients will be enrolled. Participants will be randomized into 1:1 allocation, blocked randomization with 30 participants in the vitamin D group and 30 participants in the placebo group. The randomization will be done using a computer randomization program before the intervention. This will be overseen by a biostatistician who is not involved in the recruitment of patients and data analysis. Hence, both participants and the research personnel are blinded until the completion of treatment.

##### Concealment mechanism {16b}

Allocation concealment will be ensured as the computer randomization programme will not release the randomization code until the patient has been recruited into the trial. Vitamin D and placebo will be dispensed to participants in visually indistinguishable forms by Clinical Research Pharmacy based on the randomization code. Hence, patients and investigators are fully blinded until the completion of treatment. Outcome assessors and statisticians are also blinded.

##### Implementation {16c}

The principal investigator will enrol participants. The computer randomization program will generate the allocation sequence at a 1:1 ratio. An independent research staff will assign participants to interventions.

### Assignment of interventions: blinding

#### Who will be blinded {17a}

Participants are blinded to the intervention. The research assistant who assists in consent seeking and monitoring of progress and adverse events will not be involved in outcome assessment. The assessor who will be another trained research assistant will be blinded to the randomization status and will not be involved in the intervention. The statistician responsible for the randomization is not involved in other parts of the study including data analysis.

#### Procedure for unblinding if needed {17b}

In normal circumstances, the blinding will be maintained unless a serious adverse event occurs. Unblinded participants will then exit the trial, and the medical conditions will be managed accordingly. The management results will be recorded on the clinical report form and reported to the Joint Clinical Research Ethics Committee of the Chinese University of Hong Kong and the New Territories East Cluster of the Hospital Authority.

### Data collection and management

#### Plans for assessment and collection of outcomes {18a}

##### Anthropometric measurement

Body mass index (BMI) would be calculated by the measured height and weight. We would measure the waist circumference as well. Subcutaneous fat would be measured using skinfold techniques for the triceps brachii and biceps brachii.

##### Biochemical assays

Blood samples will be taken under non-fasting conditions. The serum obtained (5 ml) will be immediately stored at − 80 °C until analysis. Quantitative analysis for serum 25(OH) Vit-D assay will be performed using LC-Qtrap/MS.

##### Isokinetic muscle strength assessment

The dynamometer (Biodex System 4, Biodex Medical Systems Inc., New York, USA) will be used. Prior to the test, the subjects will engage in a standardized warm-up exercise consisting of 5 min of cycling. The knee extension and flexion will be tested in concentric and concentric contractions at 60°/s and 180°/s [2]. Subjects will be seated on the dynamometer chair with their hips flexed to 85°. The speed of 180°/s is chosen to perform the fatigue test since high-speed workouts are expected to tire the fast-twitch muscle fibres more quickly [17]. For calculating peak torque, the single highest value within the 30 repetitions will be considered. The fatigue test will reflect muscle endurance, and the trends for peak torque, work, and power will be analysed. To calculate the percentage decrease for each variable, the FI (fatigue index) will be used [14]. The formula for percentage decrease is 100 − [(last 5 repetitions/first 5 repetitions) × 100]. If an individual fails to achieve their peak torque within the first 3 repetitions, a second F.T. will be calculated using the formula: Per cent decrease = [100 − [(last 5 repetitions/highest consecutive 5 repetitions) × 100]. The highest consecutive five repetitions will be determined by values attained from the repetitions immediately before and following the single highest repetition value [14]. The re-test reliability has been proven [18].

##### Isometric muscle strength assessment

After warming up on a stationary bicycle for 5 min, the strength of the quadriceps muscle will be measured using a Biodex dynamometer (Biodex System 4, Biodex Medical Systems Inc., New York, USA) through maximal voluntary isometric contractions (MVIC). The uninjured limb will be tested first, followed by the injured limb. To isolate knee movement, the participants will be stabilized with straps placed over the trunk, pelvis, and thigh, with the hip flexed at 90° and the knee flexed at 45°, respectively [11]. To get the participants accustomed and warmed up, three sub-maximal voluntary contractions will be performed. Afterward, the participants will be instructed to perform three 5-s MVICs, with a 30-s rest period between each contraction. During the contractions, participants will be motivated to “kick as fast and hard as possible” verbally. The highest peak torque achieved among the three contractions will be collected as the MVIC and normalized by body mass for analysis.

The quadriceps rate of torque development (RTD) will be obtained from the MVIC test. The early and late RTD values will be calculated by determining the average slope of torque versus time curve measured from 0 to 50 ms (RTD0-50) and 100 to 200 ms (RTD100-200), respectively, after the onset of MVIC [8]. The onset of a contraction is identified as the torque ≥ 20 Nm. The highest RTD will be selected and normalized to body mass for analysis.

The superimposed burst technique (SIB) will be used to measure activation failure. This technique delivers a series of electrical stimulations to the quadriceps during a MVIC, causing a transient increase in muscle torque. All participants will be given a minimum of 5 min of rest after completing the MVIC test to prevent muscle fatigue. The position of SIB is the same as the MVIC test on the Biodex dynamometer. Two self-adhesive electrodes [ValuTrode (7.5 × 13 cm), Axelgaard manufacturing, CA, USA] will be attached to the quadriceps along the femoral nerve. All participants will be instructed to perform three additional 5-s quadriceps MVICs. A 1-min rest interval is provided between each contraction. During the MVIC of the quadriceps, an electrical stimulator (DS7R; Digitimer, Welwyn Garden City, UK) controlled by the Signal 7.05a software (CED Software, Cambridge, UK) will automatically deliver a supramaximal electrical stimulus. The stimulus consisted of 10 pulses with a pulse duration of 600 μs, delivered at a rate of 100 pulses per second. This occurred at the 3rd second of the MVIC. The intensity of the supramaximal electrical stimulus is determined prior to the SIB test. Electrical stimulations will be progressively delivered to the quadriceps, increasing by 100 mA each time until the stimulation-induced torque reached a plateau at rest. To ensure full stimulation of the quadriceps, the SIB test utilizes a current intensity of 120% plateau. In order to ensure maximal participant exertion, a successful trial is defined as achieving greater than 90% MVIC of quadriceps torque prior to the electrical stimulation. To represent full activation of the quadriceps, a central activation ratio (CAR) will be utilized. The following formula is used to calculate CAR: CAR = MVIC/(MVIC + stimulation provoked torque (SPT)). For analysis, the highest CAR obtained from three successful trials was recorded.

##### Radiological assessment

Ultrasound imaging: The muscle thickness of the vastus medialis (VM), vastus lateralis (VL), and rectus femoris (RF) on both the injured and uninjured leg will be measured using the Aixplorer® ultrasound system (SuperSonic Imagine, Aix-en-Provence, France) and a linear transducer probe with a bandwidth of 2–10 MHz (SuperLinear™ SL10-2, Vermon, Tours, France). The participants will lie on a treatment table in a supine position during the assessment. A measuring tape will be used to locate VM, VL, RF, and the patella by palpation, and then marked with a pen for reference. Following the guidelines below, we consistently measure and label the locations as the three muscle groups for ease of comparison across patients. The following are the locations of specific points on the leg: RF is located at half of the distance from the anterior superior iliac spine (ASIS) to the superior pole of the patella, VM is located at one-fifth of the distance away from the midpoint of the medial patella border to the ASIS, and VL is located at one-third of the distance from the midpoint of the lateral patella border to the ASIS. After locating the anatomical points, excess contact gel will be applied to these points. The transducer probe will be aligned in the transverse plane and moved along the entire muscle bundle to capture a view of the VM, VL and RF. The operator will position the probe into the sagittal plane to measure muscle thickness upon the marked anatomical points. Minimal pressure will be applied to the limb to prevent muscle deformation. The results will be derived from three measurements averaged.

HR-pQCT: The HR-pQCT (ExtremCT II, Scanco, Switzerland) will be used to measure the size of the bone shell at the graft tunnel interface in the injured knees. The scan will consist of a total of 1344 axial slices with an image matrix of 2304 × 2304, taken at a nominal isotropic voxel size of 60.7 μm. The scan region will be defined by the scout view image that is acquired in the sagittal plane. The total scanning length will be 81.6 mm, covering the tunnel from the proximal tibia to the distal femoral condyle. The X-ray settings used will be 68 kVp, 1470 μA, 100 ms integration time, and 156 mAs per stack (168 slices). To avoid artefacts, patients will be instructed to sit still during the measurement. The image segmentation will be performed to select the bone shell features at graft tunnel interfaces near the femoral and tibial intra-articular apertures. This will be done using standardized threshold values at a thickness of approximately 2.5 mm (40 slices). The geometric transformation will be used to adjust for variations in the angle between the scanning axis and the tunnel axis by using a cosine function. The bone shell size will be presented as a summation of the segmented angle of incidence (AOI) of 40 slices to yield a volume of interest (VOI) in μm^3^.

##### Passive knee laxity

To measure the anterior–posterior knee laxity, the KT-1000 knee ligament arthrometer (MEDmetric Corp, San Diego, CA, USA) will be used. A manual force test will be applied until a 30-lb sound signal is activated. Three trials will be performed.

##### Biomechanics-motion analysis

The lower-body marker set-up will be used to assess kinematics via a skin marker-based motion analysis system (Vicon MX, Oxford, UK) following the OSTRC standard protocol, utilizing 16 cameras and 16 reflective skin markers. The kinetic variables including vertical and horizontal ground reaction force (GRF) and joint moments will be measured using a synchronized force plate (0.60 × 0.40 m, model OR6-7, AMTI, Watertown, MA) at the centre of the capture volume at 1000 Hz.

Single-leg hop (SLH) task: The SLH test will be performed as reported in the previous studies [19]. Three trials will be performed on each leg followed by familiarization. The SLH test will be considered valid if the patients can hop the maximum distance while keeping their balance for at least 2 s after landing.

Single leg squat (SLS) task: The subjects will begin by standing upright with their toes pointed forward and then squat down at their own pace. Once they reach the designated flexion angle, they will be asked to hold the position for ten seconds. If the subject is unable to maintain their balance, the trial will be deemed invalid. All participants will practice enough to achieve the required knee angle, which is between 40 and 50 degrees.

##### Self-reported outcomes

Lysholm Score: Lysholm Knee Score is a questionnaire that examines knee-specific symptoms and function of daily living. It consists of eight items, with a total score ranging from 0 to 100 and a higher score indicates a better outcome with fewer symptoms of disability.

International Knee Documentation Committee Subjective Knee Form (IKDC): IKDC is a self-reported questionnaire that measures symptoms, knee function and activity of daily living. The questionnaire consists of 10 questions, with a total score ranging from 0 to 100 and a higher score indicates greater knee function.

Tegner Score: The Tegner Activity Scale will be used to assess activity levels related to sports on a scale of 0 to 10. Zero represents a low activity level, and 10 represents the highest activity level.

Physical Activity Questionnaire: The level of physical activities during the past year will be evaluated with a validated Chinese version of the quantitative physical activity questionnaire adapted from Baecke et al. [9].

Food Frequency Questionnaire: The level of estimated vitamin D level taken from food will be evaluated with a validated Chinese version of the food frequency questionnaire.

Sunlight Exposure Questionnaire: The level of estimated vitamin D level absorbed from sunlight exposure will be evaluated with a validated Chinese version of the sunlight exposure questionnaire.

The assessment schedule is shown in Table 1.

**Table 1:** Assessment schedule

Assessment	Baseline	1st follow-up	2nd follow-up	2 months post-intervention
Anthropometric measurement	✓	✓	✓	✓
Isokinetic assessment	✓	✓	✓	✓
Isometric assessment	✓	✓	✓	✓
Ultrasound imaging of muscle thickness	✓	✓	✓	✓
Serum vitamin D evaluation	✓	✓	✓	✓
Passive Knee laxity by KT-1000	✓	✓	✓	✓
Reaming size of the bone by XtremeCT	✓	✓	✓	✓
Ground reaction force	✓	✓	✓	✓
Knee joint moments	✓	✓	✓	✓
Single-leg hop distance	✓	✓	✓	✓
Tegner Activity Score	✓	✓	✓	✓
International Knee Documentation Committee (IKDC) Questionnaire	✓	✓	✓	✓
Lysholm score	✓	✓	✓	✓
The International Physical Activity Questionnaires (IPAQ)	✓	✓	✓	✓
Adverse events		✓	✓	✓

#### Plans to promote participant retention and complete follow-up {18b}

Patients will be contacted weekly for their intake of the intervention and 1 week before the assessment to enhance the attendance rate. Special assessment session on the weekend or in the evening will be arranged under special circumstances to enhance subject compliance. Patients who default a scheduled appointment will be contacted by the investigators to re-arrange another appointment within 1 week.

#### Data management {19}

Clinical data will be collected and recorded by trained research assistants in our research centre. Clinical examination data will be entered on case report forms and then entered electronically. Consistency checks by another technician will be performed to ensure data entry accuracy. All data will be stored in password-protected computers. The study will be conducted in compliance with Good Clinical Practices to ensure the rights and well-being of the participants and that the data collected are complete and verifiable from source documents. Patients are free to withdraw from the study at any time without giving any reasons, and their medical care or legal rights will not be affected. Patient files will be maintained in storage for a period of 3 years after completion of the study.

#### Confidentiality {27}

All personal information and consents on enrolled patients collected on paper versions will be kept in locked units at the participating practice and later at the coordinating centre to be archived. Each patient will be assigned an identification code. All information collected and inputted on the electronic database will be based on the identification code and therefore does not contain any personalized information that enables the identification of the patient. The document containing the information on the identification code and the identity of the patient will be kept separate from the study data files and data sheets. The patient identification code list and database can only be retrieved by dedicated study team members or be inspected by study monitors for quality checking and verification.

#### Plans for collection, laboratory evaluation, and storage of biological specimens for genetic or molecular analysis in this trial/future use {33}

Blood will be collected at the clinical sites for evaluation. After arrival at the local research laboratory at each site, the samples will be collected, transported, stored, and prepared according to local protocols. Blood will be stored at 2–8 °C before handling within the required time. All samples collected during the trial will be labelled with the patients’ identification code and will not contain any identifiable data. Patients have the option of consenting to their samples being stored for future research uses. Samples will be stored anonymously at a central location for a minimum of 5 years and a maximum of 10 years after completion of the study, after which these specimens will be destroyed by incineration according to local guidelines and protocols. The potential usage of the stored samples is included in the informed consent form. Nevertheless, further usage of the samples will need to be approved by the institutional ethical committee.

## Statistical methods

### Statistical methods for primary and secondary outcomes {20a}

Statistical analysis will be performed using the SPSS software (SPSS 26.0). The normality of the data will be tested using the Kolmogorov–Smirnov test. A repeated-measures one-way analysis of variance (ANOVA) will be used to compare quadriceps muscle strength (isokinetic assessment), serum myokine levels, and results of questionnaires at the time points. A non-parametric Mann–Whitney *U* test will be used to compare questionnaire results (ordinal data) between the intervention and placebo groups.

### Interim analyses {21b}

Interim analysis will be performed when approximately 10% of our sample have completed follow-up assessments. The preliminary findings will be presented in conference to promote our study.

### Methods for additional analyses (e.g. subgroup analyses) {20b}

Additional analyses will include subgroup analyses to estimate treatment effects for both female and male participants.

### Methods in analysis to handle protocol non-adherence and any statistical methods to handle missing data {20c}

We will generally perform the analysis using the intention-to-treat (ITT) at the subject level for each outcome. All the participants with a recorded outcome will be included in the analysis according to the intervention group to which they have been randomized. Additionally, we will take the cluster-randomized crossover design effect into consideration. Adjustment for multiple comparisons among interventions will be used. To assess the risk of bias related to orthopaedic wards that have not completed patient recruitment, multiple imputations will be performed for the primary outcome and presented as sensitivity analyses. At the subject level, when a variable is missing, we will assume that most likely the missing variable has a normal or mean value.

### Plans to give access to the full protocol, participant-level data, and statistical code {31c}

The protocol has been uploaded on ClinicalTrials.gov (ID: NCT05174611). The data of this study will also be available from the principal investigator upon reasonable request.

### Oversight and monitoring

#### Composition of the coordinating centre and trial steering committee {5d}

The principal investigator is responsible for the design of the study and the coordination of different cooperation partners. The research team comprises the trial steering committee responsible for the recruitment, assessments, output delivery, and data analysis. The patients after cruciate ligament reconstruction who fulfil the inclusion criteria will be invited to join the study. However, there will be no public involvement.

#### Composition of the data monitoring committee, its role, and reporting structure {21a}

The principal investigator and co-investigators will monitor the data collection and storage to ensure that the data is kept and used in accordance with the protocol. A statistician, who is independent from the sponsor and any competing interests, will be responsible to inspect clinical data collected during the study period, review the interim analysis, and report back to the investigators for any action required. The data monitoring committee is not considered as PEMF is a low-risk intervention.

#### Adverse event reporting and harms {22}

Although vitamin D is presumed safe, adverse events will be checked by the investigators at every follow-up visit. Any adverse event, whether they are related to the study or not, will be recorded in the adverse effect report form provided by the Hong Kong Hospital Authority using standard adverse event language. A serious adverse event will be reported to the Hong Kong Hospital Authority Research Ethics Committee within 24 h of the event. The principal investigator will be responsible to follow the management of the serious adverse event until resolution or conclusion. The investigators and the trial steering committee will determine whether an adverse event or serious adverse event is related to the study intervention. The intervention will be stopped immediately if the reported event is related, with a referral to seek medical attention provided by the principal investigator.

#### Frequency and plans for auditing trial conduct {23}

As per the university’s requirement, an independent auditor will conduct the annual review throughout the project period.

#### Plans for communicating important protocol amendments to relevant parties (e.g. trial participants, ethical committees) {25}

There is no plan for modifying the protocol at this juncture. However, any amendment to the protocol will be submitted by the principal investigator to be approved by the research grant committee of the Direct Grant and the ethical committee before implementation. In addition, the trial participants will also be notified as well.

#### Dissemination plans {31a}

The research findings will be published in peer-reviewed journals and disseminated to healthcare professionals, the public, and other relevant groups as soon as the results are available. The funder has no role or restriction in the decision of publication.

## Discussion

The primary goals of ACLR are to restore knee function in patients who have suffered from ACL injury and enable them to RTP. However, patients commonly experience persistent weakness in their quadriceps muscle after undergoing ACLR, and a significant percentage of them (35%) are unable to achieve RTP [8]. The weakness of the quadriceps muscles can negatively affect athletic performance and increase the risk of re-injury. Vitamin D has long been recognized for its musculoskeletal effects. Patients with low vitamin D levels had greater quadriceps fibre cross-sectional area loss after ACLR compared to those with normal or higher vitamin D levels [20]. Our study proposes using vitamin D to promote muscle gains in patients experiencing persisting muscle weakness after ACLR.

Our study may bring various benefits to patients after ACLR. After an ACL injury, patients experience a decline in neuromuscular function, which lasts even after the operation [15]. Vitamin D has been shown to play a role in improving neuromuscular function. Vitamin D supplementation may lead to improved neuromuscular function in older adults, potentially aiding in the prevention of falls [7, 16]. The presence of vitamin D receptors in the skeletal muscle tissue and their role in muscle strength and coordination further support the link between vitamin D and neuromuscular function [5]. Therefore, correcting vitamin D status may have potential benefits for enhancing neuromuscular function in individuals, including patients after ACLR.

Our study design has strengths. Firstly, the format of Clinical Research Pharmacy as randomized clusters will enhance the fidelity of the interventions. Second, dispensing supplements in two installments will enhance the subject compliance and prevent a high drop-out rate. Thirdly, two questionnaires will be used to estimate daily vitamin D intake and eliminate its effect. However, there is a limitation to this study design. Patient recruitment with inconsistent post-operative time intervals may result in biased outcomes.

## Trial status

The protocol version 1 was dated 08 December 2022, and version 7 was dated 06 March 2023. Reasons for revision are described in {3}.

Study recruitment started on 15 March 2023, and the recruitment is still ongoing. The recruitment will finish on 1 March 2025, and the completion of the trial remains scheduled on 1 September 2025.

### Supplementary Information

**Supplementary Material 1.**

## Data Availability

Any data required to support the protocol can be supplied on request.

## Abbreviations

ACLR: Anterior cruciate ligament reconstruction
RTP: Return to play
ACL: Anterior cruciate ligament
GMP: Good Manufacturing Practice
FI: Fatigue index
RTD: Rate of torque development
CAR: Central activation ratio
BMI: Body mass index
MVICs: Maximal voluntary isometric contractions
SIB: Superimposed burst technique
SPT: Stimulation provoked
VM: Vastus medialis
VL: Vastus lateralis
RF: Rectus femoris
ASIS: Anterior superior iliac spine
AOI: Segmented angle of incidence
VOI: Volume of interest
GRF: Ground reaction force
SLH: Single-leg hop
SLS: Single-leg squat
IKDC: International Knee Documentation Committee Subjective Knee Form
ITT: Intention-to-treat
